# A chromosome-level genome assembly of the Peruvian Algarrobo (*Neltuma pallida*) provides insights on its adaptation to its unique ecological niche

**DOI:** 10.1093/g3journal/jkae283

**Published:** 2024-12-05

**Authors:** Renato La Torre, John P Hamilton, Manuel Saucedo-Bazalar, Esteban Caycho, Brieanne Vaillancourt, Joshua C Wood, Manuel Ramírez, C Robin Buell, Gisella Orjeda

**Affiliations:** Laboratory of Genomics and Bioinformatics for Biodiversity, Faculty of Biological Sciences, Universidad Nacional Mayor de San Marcos, Lima 15081, Peru; Center for Applied Genetic Technologies, University of Georgia, Athens, GA 30602, USA; Department of Crop and Soil Sciences, University of Georgia, Athens, GA 30602, USA; Laboratory of Molecular Biology, Department of Biology and Biochemistry, Universidad Nacional de Tumbes, Tumbes 24001, Peru; Laboratory of Genomics and Bioinformatics for Biodiversity, Faculty of Biological Sciences, Universidad Nacional Mayor de San Marcos, Lima 15081, Peru; Center for Applied Genetic Technologies, University of Georgia, Athens, GA 30602, USA; Center for Applied Genetic Technologies, University of Georgia, Athens, GA 30602, USA; Laboratory of Genomics and Bioinformatics for Biodiversity, Faculty of Biological Sciences, Universidad Nacional Mayor de San Marcos, Lima 15081, Peru; Center for Applied Genetic Technologies, University of Georgia, Athens, GA 30602, USA; Department of Crop and Soil Sciences, University of Georgia, Athens, GA 30602, USA; Institute of Plant Breeding, Genetics, and Genomics, University of Georgia, Athens, GA 30602, USA; The Plant Center, University of Georgia, Athens, GA 30602, USA; Laboratory of Genomics and Bioinformatics for Biodiversity, Faculty of Biological Sciences, Universidad Nacional Mayor de San Marcos, Lima 15081, Peru

**Keywords:** Algarrobo, carob, Mesquite, *Prosopis*, nanopore, genome assembly, annotation

## Abstract

The dry forests of northern Peru are dominated by the legumous tree *Neltuma pallida* which is adapted to hot arid and semiarid conditions in the tropics. Despite having been successfully introduced in multiple other areas around the world, *N*. *pallida* is currently threatened in its native area, where it is invaluable for the dry forest ecosystem and human subsistence. A major tool for enhancing ecosystem conservation and understanding the adaptive properties of *N*. *pallida* to dry forest ecosystems is the construction of a reference genome sequence. Here, we report on a high-quality reference genome for *N. pallida*. The final genome assembly size is 403.7 Mb, consisting of 14 pseudochromosomes and 63 scaffolds with an N50 size of 26.2 Mb and a 34.3% GC content. Use of Benchmarking Universal Single Copy Orthologs revealed 99.2% complete orthologs. Long terminal repeat elements dominated the repetitive sequence content which was 51.2%. Genes were annotated using *N. pallida* transcripts, plant protein sequences, and ab initio predictions resulting in 22,409 protein-coding genes encoding 24,607 gene models. Comparative genomic analysis showed evidence of rapidly evolving gene families related to disease resistance, transcription factors, and signaling pathways. The chromosome-scale *N. pallida* reference genome will be a useful resource for understanding plant evolution in extreme and highly variable environments.

## Introduction

The Peruvian algarrobo, *Neltuma pallida* (Humb. & Bonpl. ex Willd.) C.E.Hughes & G.P.Lewis (Fabaceae: Caesalpinioideae), is a tree legume native to arid and semi-arid areas along the Pacific coast in northwestern South America (Burkart 1976; Hughes *et al.* 2022). *N. pallida* is the dominant tree species in tropical dry forests of Peru and Ecuador, adapted to the marked patterns of long dry and short rainy seasons, persistent warm temperatures, and high salinity soil that characterize these fragile ecosystems (Zanoni and Zeña 1998; Leal-Pinedo and Linares-Palomino 2005; Grados *et al.* 2022). *N. pallida* provides multiple ecological services and has high ecological importance, as it enables the survival of other wild species, increases soil fertility, and is used by local communities for consumption, livestock forage, and wood (Depenthal and Yoder 2018; Dostert *et al.* 2013).

Algarrobo has been introduced and naturalized around the world outside its native area and is considered an invasive species in some countries (Pasiecznik *et al.* 2001; Gallaher and Merlin 2010; Shackleton *et al.* 2014). The ability of *N. pallida* to thrive in dry environments is hypothesized to be a consequence of morphological and physiological adaptations, as well as high phenotypic plasticity (Castillo *et al.* 2021; Salazar *et al.* 2021). However, despite its success in shaping the dry forests, *N. pallida* is currently threatened by multiple abiotic and biotic stresses in its native area and urgent conservation efforts are needed (Cuentas and Salazar 2017; Whaley *et al.* 2019). Current threats include illegal logging, changes in land use, intensive livestock rearing, the emergence of pests such as insects and fungi that result in more disease, and climate variability (Hocquenghem 1998; Beresford-Jones *et al.* 2009; Whaley *et al.* 2020; Salazar *et al.* 2021; Ewens *et al.* 2022). Recently, the species has been targeted by law as a national priority for research, conservation, and protection by the Peruvian government.

The species *N. pallida* was formerly classified under the section Algarobia from the genus *Prosopis*, which also included sections Strombocarpa, Monilicarpa, Prosopis, and Anonychium (Burkart 1976). A recent study supported the disintegration of the former *Prosopis* genus into several genera: *Neltuma* (former sections Algarobia and Monilicarpa), *Prosopis*, *Anonychium,* and *Strombocarpa* (Hughes *et al.* 2022). All these species are diploid (2*n* = 28) except for the tetraploid *N. juliflora*, are mainly distributed in America, and have often shown to interbreed (Burkart 1976; Pasiecznik *et al.* 2001; Trenchard *et al.* 2008). Most available haploid genome size estimates for American accessions are among the smallest in legumes (396.6–481.2 Mbp), including *N. pallida* (408.8 Mb) (Bukhari 1997; Sliwinska *et al.* 2009); although a recent study estimated a genome size of 707.2 Mb for *N. alba* (Kong *et al.* 2023). On the other hand, some non-American and introduced accessions have had larger estimates (579.3–691.9 Mbp) and a degree of both within-individual and within-population genome variability likely due to hybridization (Bukhari 1997; Bennett and Leitch 2005; Mazibuko 2012; Sudalaimuthuasari *et al.* 2022).

One tool for understanding species evolution and adaptation to their environments, characterizing and understanding population structures, and revealing genes underlying adaptive traits is the construction of a reference genome sequence (Theissinger *et al.* 2023). Previous studies have reported chloroplast assemblies for closely related species such as *S. tamarugo*, *P. cineraria*, *P. farcta*, *N. juliflora,* and *N. glandulosa*, as well as for *N. pallida*, but the scope of plastid sequences is limited compared to nuclear genomes (Asaf *et al.* 2020; Contreras-Díaz *et al.* 2021; Caycho *et al*. 2023). On the other hand, while there are genome sequences for *N. alba* (Kong *et al.* 2023) and *P. cineraria* (Sudalaimuthuasari *et al.* 2022), these are fragmented, and a chromosome-scale reference assembly for the group is lacking.

In this study, we report the construction and analysis of a chromosome-scale reference genome sequence for *N. pallida*. Our aim is to enable genomic studies to understand the mechanisms underlying *N. pallida* adaptive traits and facilitate conservation through genetic and biotechnology approaches.

## Methods and materials

### Tissue source and collection methods

**Fig. 1.**
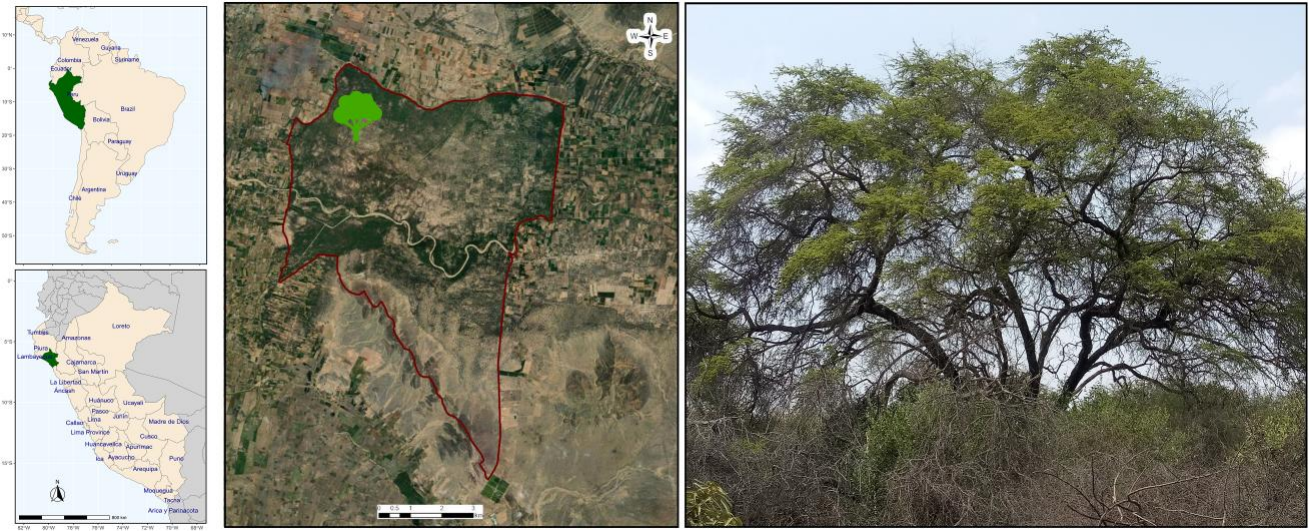
Sampling site of a representative algarrobo, *N. pallida*, tree at the national protected area Historic Sanctuary Bosque de Pómac, Lambayeque, Peru. Coordinates are 6°26′39.4″ S and 79°48′16.6.6″ W.

A healthy wild adult individual *N. pallida* tree was selected based on previous screening in the Historic Sanctuary Bosque de Pómac, a protected area in Lambayeque, Peru, that contains the largest and most representative *N. pallida* natural population (Fig. 1). Diverse tissues from the same individual were collected in the field for DNA and RNA extraction. Leaves for DNA extraction and high-throughput chromosome conformation capture (Hi-C) library preparation were collected and stored fresh in bags containing silica gel. Leaves, petioles, axillary buds, flowers, fruits, roots, and branches for RNA extraction were harvested and immediately stored in liquid nitrogen. Physical voucher specimens from the selected individual were deposited in the Museo de Historia Natural—UNMSM herbarium with the collection code USM no 335439 in Lima, Peru. Additional pictures of the diverse tissues collected in this study can be obtained from a previous publication (Caycho *et al.* 2023).

### DNA extraction and sequencing

Genomic DNA for short-read sequencing was extracted from leaves using a CTAB-based protocol (Doyle 1991). An Illumina TruSeq DNA PCR Free library with a target insert size of 350 bp was constructed and sequenced on a NovaSeq 6000 instrument in paired-end mode (150 nt). High-molecular weight DNA for long-read sequencing was extracted from leaves as described previously (Vaillancourt and Buell 2019), and two Oxford Nanopore Technologies (ONT) ligation sequencing (SQK-LSK110) libraries were prepared and sequenced on ONT FLO-MIN106D flow cells using a MinION Mk1C. The ONT long reads were base called using the super high accuracy (SUP) model with Guppy v.6.3.7. Two Hi-C libraries were prepared by Phase Genomics Inc. (Seattle, WA) from fresh leaf cells using the Proximo Hi-C Plant v4.0 kit and protocol with a modified fragmentation enzyme cocktail containing DpnII, Ddel, Hinfl, and MseI (Bickhart *et al.* 2017). Libraries were sequenced by Phase Genomics on an Illumina NovaSeq 6000 (San Diego, CA) in paired end mode to 150 nt.

### RNA extraction and cDNA sequencing

Total RNA was extracted using the Quick-RNA MiniPrep kit (Zymo Research) using up to 200 mg of plant material. Two multiplexed ONT PCR-cDNA libraries were prepared using the SQK-PCB109 (ONT) kit, one including cDNA from petioles, axillary buds, roots, and branches in triplicate, and the other from flowers, fruits, leaves, and blank in triplicate. cDNA libraries were sequenced on FLO-MIN106D flow cells with a MinION Mk1C. ONT cDNA reads were base called using the super high accuracy (SUP) model in Guppy v.6.3.7.

### Genome assembly and polishing

**Fig. 2.**
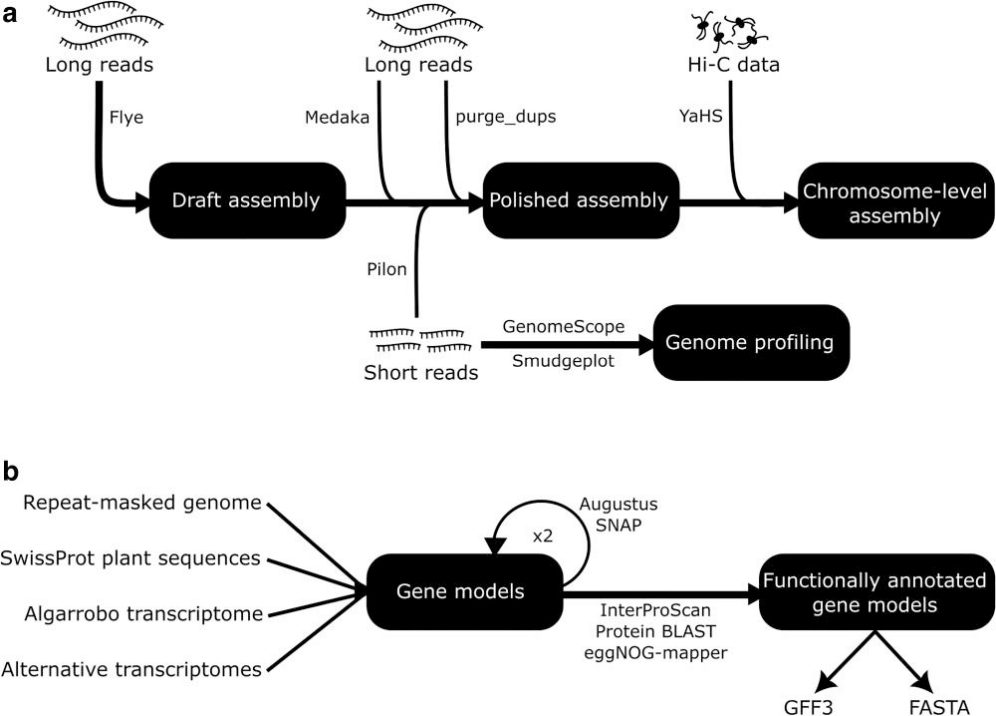
Procedure for the genome profiling, assembly, polishing, and scaffolding a) and annotation steps b) followed in this study. The amount of input data for the genome assembly is detailed in Table 1.

Sequencing data and the process used for genome profiling, assembly, polishing, and scaffolding are summarized in Fig. 2a, Table 1, and available under National Center for Biotechnology Information (NCBI) Project number PRJNA1102382. K-mer spectrum analysis and genome profiling were performed with a k-mer size of 21 using short reads input into KAT version 2.4.2, Smudgeplot version 0.2.5, and Genomescope 2.0 version 1.0.0 (Mapleson *et al.* 2017; Vurture *et al.* 2017; Ranallo-Benavidez *et al*. 2020). ONT reads longer than 10 kb and with an average quality score greater than 9 were assembled using Flye version 2.9.1 with the option –nano-hq (Kolmogorov *et al.* 2019) to generate an initial draft genome. The assembly was polished twice using Medaka version 1.11.3 (https://github.com/nanoporetech/medaka) with the setting −m r941_min_sup_g507, and twice using Pilon version 1.24 configured for a diploid organism (−diploid) to fix only SNPs and indels (−fix bases) (Walker *et al.* 2014). Contigs shorter than 50 kb were removed, and the assembly was filtered using purge_dups version 1.2.6 to resolve haplotigs and contig overlaps (Guan *et al.* 2020). To construct a chromosome-scale assembly, the Hi-C reads were aligned to the contigs using BWA-MEM version 0.7.17 (Li 2013) with the parameters (−5SP) and duplicates marked with samblaster version 0.1.26 (Faust and Hall 2014). The contigs were then scaffolded with YaHS version 1.2a.2 (Zhou *et al.* 2023). The resulting chromosome-scale assembly was inspected with Juicebox version 2.20.00 (Durand *et al.* 2016) and the final assembly was exported from YaHS. Lastly, the genome was screened for contaminants and adapters using the NCBI FCS tool version 0.5.4 (Astashyn *et al.* 2024). At each stage, the quality of the assembly was assessed using Benchmarking Universal Single-Copy Orthologs (BUSCO) version 5.7.0 with the embryophyta_odb10 dataset (Manni *et al.* 2021).

**Table 1.** Summary of the data used for assembling the genome of *N. pallida*.

Type of data	Reads (*n*)	Total yield (bp)	Read length (nt)	Sequencing depth (X)*^a^*
Short reads (Illumina)	226,347,312	68,356,888,224	151	169.2
Long reads (Nanopore)	1,002,522	19,200,665,956	20,132 (N50)	47.5
Hi-C	189,884,148	56,965,244,400	150	141.0

^*a*^Based on the total length of the final assembly.

### Genome annotation

The annotation steps taken in this study are summarized in Fig. 2b. A custom repeat library was constructed using RepeatModeler version 2.0.5 (Flynn *et al.* 2020) and LTR_retriever version 2.9.0 (Ou and Jiang 2018) and then used to mask the final assembly using RepeatMasker version 4.1.5 (https://github.com/Dfam-consortium/RepeatMasker). For assembling the transcriptome, ONT cDNA reads were filtered using porechop version 0.2.4 (https://github.com/rrwick/Porechop) and NanoFilt version 2.8.0 (De Coster *et al.* 2018) to trim adapters and filter reads by length (l = 500 bp) and quality (*q* > 9), before being mapped to the genome assembly using minimap2 version 2.28 (Li 2018). Transcriptome assemblies for all *N. pallida* tissues were constructed with StringTie version 2.2.3 (Shumate *et al.* 2022). The annotation was performed using MAKER version 3.01.04 (Campbell, Law et al. 2014) with the repeat-masked genome, SwissProt plant sequences, and reference-guided *N. pallida* transcriptome as empirical evidence, and leaf transcriptomes of *N. alba* (GAOO00000000.1) and *P. cineraria* (GFOW00000000.1) as alternative transcript evidence (Fig. 2b). Gene models based on empirical evidence with an annotation edit distance (AED) up to 0.25 and coding proteins longer than 50 aa were used to train the ab initio gene prediction programs SNAP version 2006-07-28 and Augustus version 3.5.0 (Korf 2004; Stanke *et al.* 2006). Augustus was trained using BUSCO with the embryophyta_odb10 dataset and the Arabidopsis HMM model optimized for the *N. pallida* genome (−long). A second MAKER run was performed using the annotations from the first round and the gene prediction software. The gene models were filtered to retrain SNAP and Augustus as described above for a third maker run in which gene models from alternative splicing were kept. Gene models containing masked regions within the coding sequence (CDS) were removed. Gene models without external evidence but encoding a known functional domain as identified by InterProScan version 5.65-97.0 (Jones *et al.* 2014) were retained (standard maker build) (Campbell, Holt *et al.* 2014). Functional annotation was assigned using InterProScan, BLAST version 2.14.1 + searches against the SwissProt plant database (Altschul *et al.* 1990), and EggNOGmapper version 2.1.12 with the taxonomic scope to Viridiplantae (Huerta-Cepas *et al.* 2019; Cantalapiedra *et al.* 2021; Buchfink *et al.* 2021).

### Comparative genomics

A multi-species comparative analysis was performed using the proteomes of *N. pallida* and other plant species including *N. alba*, *P. cineraria*, *Senna tora*, *Bauhinia variegata, Medicago truncatula, Glycine max, Prunus persica,* and *Vitis vinifera* (Supplementary Table 1). For all species, proteins from all chromosomes/scaffolds were used with only one representative isoform per protein-coding gene being included in the analyses. For the synteny analysis, collinear blocks were determined through two-way comparisons using the MCscan pipeline (in JCVI utility libraries version 1.4.2) (Tang *et al.* 2008). Single-copy orthogroups covering all species were inferred using OrthoFinder version 2.5.5 (Emms and Kelly 2019), aligned with mafft version 7.515 (Katoh *et al.* 2002) and trimmed with trimAl version 1.2 (Capella-Gutiérrez *et al.* 2009). The alignment was used as input for constructing a maximum-likelihood (ML) phylogenetic tree using RAxML version 8.2.12 (Stamatakis 2014) with the model JTT + I + G4 + F as determined using ModelTest-NG version 0.1.7 (Darriba *et al.* 2020). Phylogenetic hierarchical orthogroups (HOGs) were identified in a second OrthoFinder run specifying the ML tree and then filtered to discard uninformative and large (>100 genes) gene families. Shared groups were visualized using the R package UpSetR version 1.4.0 (Conway *et al.* 2017). The phylogenetic tree was calibrated by setting the separation between the outgroup and the rest of the tree at 125 mya (Wikström *et al.* 2001). This species tree was used along the filtered gene families as inputs for CAFE version 5.0.1.1 1 (Hahn *et al.* 2005) to estimate the patterns of expansion or contraction of gene families. A summary and plots of gene family dynamics were obtained using CafePlotter version 0.2.0 (https://github.com/moshi4/CafePlotter). Lastly, a gene ontology enrichment analysis was performed for the genes of rapidly expanding families using topGO version 3.18 (Alexa and Rahnenführer 2009) and the results were visualized using Revigo version 1.8.1 (Supek *et al.* 2011).

## Results and discussion

### Genome assembly and completeness

**Fig. 3.**
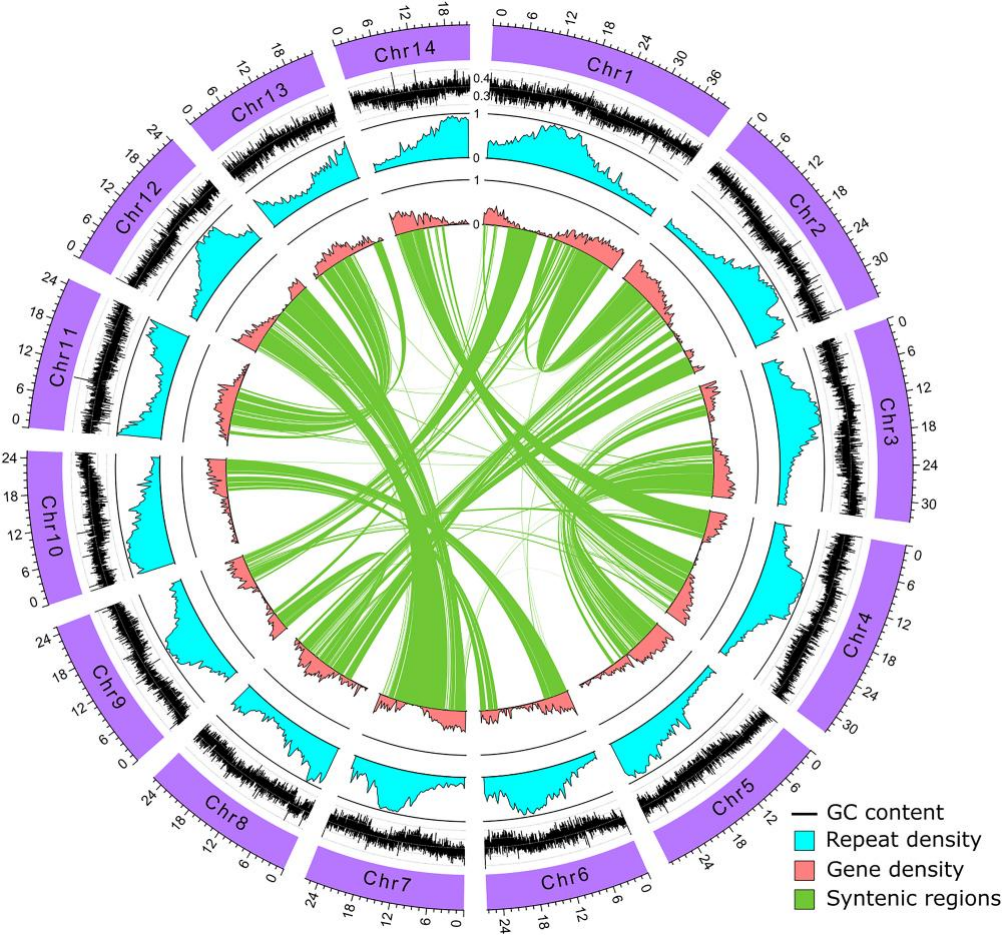
Genomic features of the *N. pallida* final assembly. Each chromosome is depicted with the axis indicating the position in Mb. GC content was calculated in 10 kb windows. Repeat and gene densities were calculated in 0.5 Mb windows.

The reference-free 21-mer spectrum analysis predicted a genome size of 442.27 Mb, 37.4% of which was repetitive and 0.574% heterozygous. Also, the smudgeplot pattern confirmed the ploidy level for *N. pallida* as diploid (Supplementary Figs. 1-3). The final *N. pallida* genome assembly size is 403.7 Mb in 14 pseudochromosomes and 63 unanchored scaffolds (Fig. 3, Supplementary Fig. 4), consistent both with the genome profiling and a previous report with flow cytometry (Bukhari 1997). The assembly has an N50 of 26.2 Mb, L50 of 7, genome-wide GC level of 34.3% and a percentage of Ns of 0.04%.

**Fig. 4.**
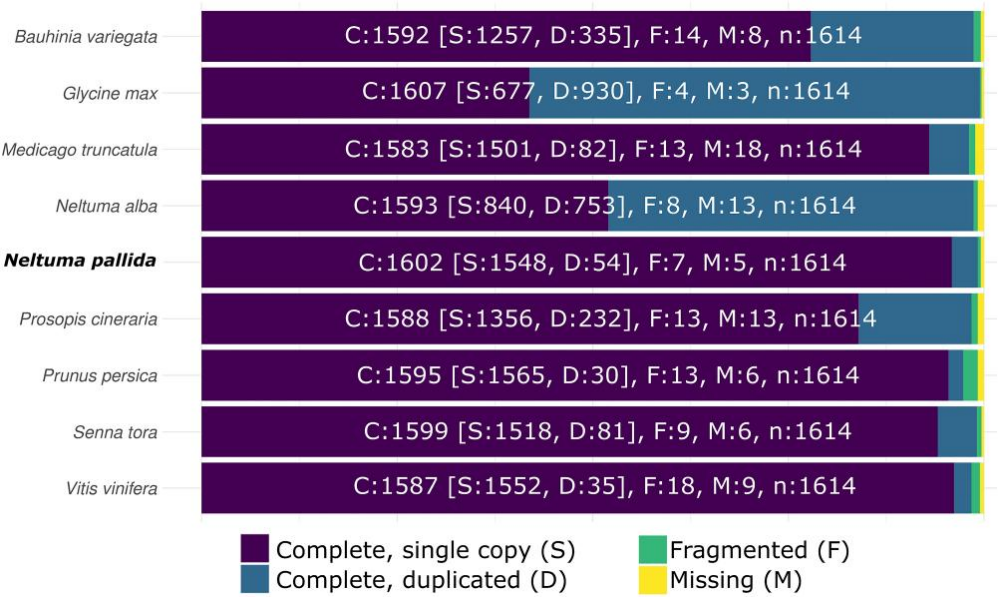
Comparison of BUSCO assessment of genome completeness among the species included in this study. The embryophyta_odb10 lineage was used.

The BUSCO completeness for the *N. pallida* assembly of 99.2% with the embryophyta lineage was comparable to those from the other species included in this study (Fig. 4). The lower number of duplicated BUSCOs in our assembly compared to those of closely related *N. alba* and *P. cineraria* likely reflect differences in the genome resolution, fragmentation and/or haplotig curation. However, further studies should evaluate whether these amount of duplicated BUSCOs are in fact signs of genomic duplications or gene expansions during the evolution of either species.

The genome of *N. pallida* ranks among the smallest among other legumes with available genome assemblies, including the closely related species *N. alba* (707.2 Mb) and *P. cineraria* (691.4 Mb) (Supplementary Table 1) (Doyle and Luckow 2003; Sudalaimuthuasari *et al.* 2022; Kong *et al.* 2023). Genome downsizing has been shown inversely associated with latitude and other environmental factors (Souza *et al.* 2019). We speculate that the reduced *N. pallida* genome could be an adaptive response to the restrictive conditions of its native area, reducing the costs of carrying and replicating large DNA amounts during the small windows of resource availability (Faizullah *et al.* 2021). Although further studies are needed to support this hypothesis, the high-quality, small, and diploid genome assembly presented here represents a valuable genomic resource for understanding the complexity of the genome evolution in *Prosopis* species and legumes in general.

### Protein-coding gene content

A total of 22,409 protein-coding genes were annotated, associated with 24,607 gene models, most of which were placed on one of the 14 pseudochromosomes (98.9%). On average, the transcript length was 3,914.46 bp and the CDS length was 1,222.89 bp, with 6.13 exons of 397.40 bp and introns of 508.12 bp long (Table 2). A total of 14,913 gene models had at least one annotated untranslated region (UTR), with an average length for the 5′ UTR of 314.17 bp and for the 3′ UTR of 500.22 bp (Table 2). The BUSCO score for the final set of *N. pallida* gene models is C: 89.1% (S: 77.6%, D: 11.5%, F: 3.2%, M: 7.7%). Most genes were supported by empirical evidence (18,762) both for exons and splice sites. The remaining genes were supported by alternative transcript sequences (transcriptomes from *N. alba* and *P. cineraria*) and/or ab initio prediction software. Most of the gene models were functionally annotated (23,881/24,607).

**Table 2.** Summary statistics from the protein-coding gene annotations for the N. pallida genome.

Description	Chromosomes	All
Length (bp)	394,713,541	403,689,856
Number of genes (*n*)	22,164	22,409
Number of gene models (*n*)	24,337	24,607
Average transcript length (bp)	3,916.81	3,914.46
Average CDS length (bp)	1,223.69	1,222.89
Average exons per transcript (*n*)	6.14	6.13
Average exon length (bp)	397.59	397.40
Average intron length (bp)	506.33	508.12
Number of gene models with UTRs (*n*)	14,795	14,913
Average 5′ UTR length (bp)	314.44	314.17
Average 3′ UTR length (bp)	500.45	500.22

As suggested by the genome size, the number of protein-coding genes for *N. pallida* is lower than the reported for species included in this study, including closely related *N. alba* and *P. cineraria* (42,275 and 30,647, respectively). Other metrics such as gene and exon length, as well as exon number for *N. pallida* are similar to those from other species (Supplementary Table 2).

### Repetitive sequences

Approximately half of the genome is composed of repetitive sequences (∼206.6 Mb, 51.2%), mostly long terminal repeats (LTR) covering 30.34% of the genome (Table 3). Other Class I retrotransposons such as long interspersed nuclear elements (LINEs) and short interspersed nuclear elements (SINEs) covered an additional 1.57% of the genome, while non-autonomous Class II transposable elements (DNA and rolling-circle TEs) covered 6.10% (Table 3). Simple tandem and low complexity repeats covered 1.71 and 0.48% of the genome, respectively. Lastly, many repetitive elements remained unclassified, covering up to 10.90% (Table 3).

**Table 3.** Repetitive element annotation of the *N. pallida* genome.

Type	Number	Total length (bp)	Genome percentage (%)
Retroelements	250,781	128,890,664	31.91
LINEs	13,314	6,005,228	1.49
SINEs	2,703	330,762	0.08
LTR elements	234,764	122,554,674	30.34
DNA transposons	59,580	21,966,440	5.44
Rolling Circle	6,908	2,666,739	0.66
Unclassified	236,044	44,046,675	10.90
Small RNA	3,135	387,624	0.10
Satellites	367	37,676	0.01
Simple repeats	166,634	6,914,633	1.71
Low complexity	40,565	1,933,635	0.48
Total		206,613,734	51.15

Similar results were reported previously for closely related species *N. alba* and *P. cineraria*, where repetitive regions covered up to 50.8 and 58%, respectively. In both cases, LTRs were also the most abundant repeat elements (20.3–41.4% for *N. alba* and 51.3% for *P. cineraria*) (Sudalaimuthuasari *et al.* 2022; Kong *et al.* 2023). Furthermore, LTRs were shown associated with an amplification of disease-resistance genes in the genome of *P. cineraria* (Sudalaimuthuasari *et al.* 2022).

### Synteny analysis

**Fig. 5.**
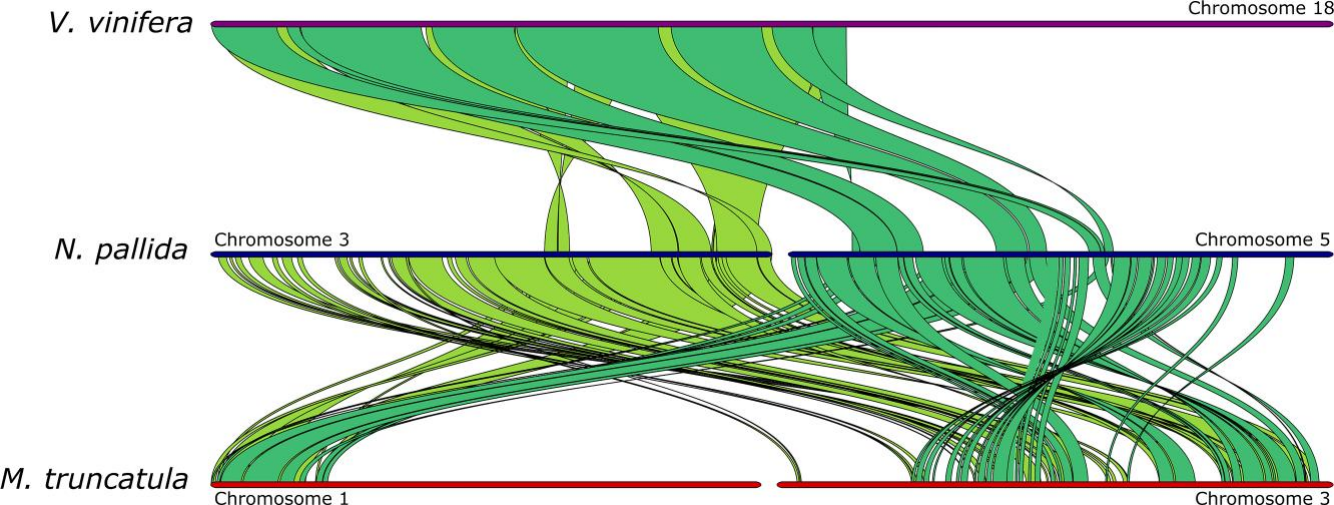
Synteny among *V. vinifera*, *N. pallida,* and *M. truncatula* chromosomes highlighting the syntenic pattern and degree of conservation of gene order among genomes despite genome-wide rearrangements.

Collinearity analyses show patterns of a whole-genome duplication (WGD) for *N. pallida*. Pairs of chromosomes within the genome of *N. pallida* shared syntenic regions (Fig. 1). Furthermore, consistent syntenic depths were found for the different between-species comparisons; for example, *N. pallida* vs. *V. vinifera* showed a 2:1 pattern and *N. pallida* vs. *M. truncatula* a 2:2 pattern (Fig. 5, Supplementary Fig. 5). These patterns likely reflect signatures of the common legume WGD in the *N. pallida* genome. As expected, the absence of the soybean-specific WGD is evident as *N. pallida* vs. *G. max* shows a 2:4 pattern (Supplementary Fig. 5). Briefly, such 2:4 pattern means 2 *N. pallida* syntenic regions for a given *G. max* genomic region, and *4 G. max* syntenic regions for a given *N. pallida* genomic region. Identical patterns were reported for the *N. alba* genome (Kong *et al.* 2023).

**Fig. 6.**
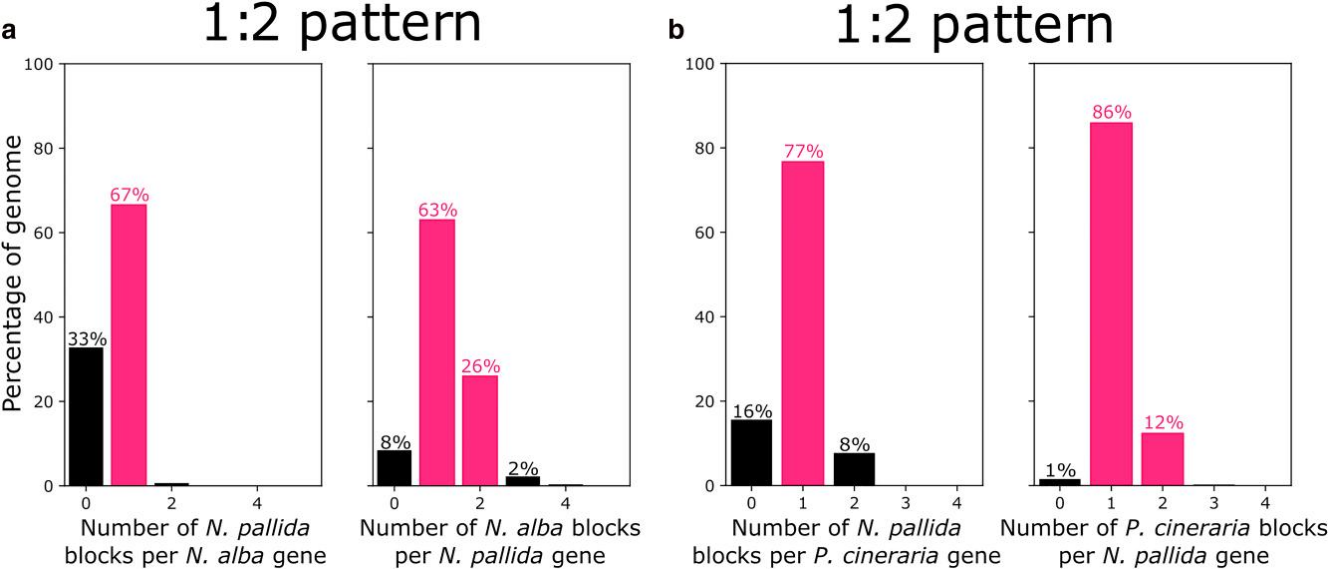
Synteny depths in pairwise comparisons between (a) *N. pallida* vs. *N. alba*, b) *N. pallida* vs. *P. cineraria*. Bars are colored to highlight the inferred pattern of synteny.

Despite their close evolutionary distance, synteny between *N. pallida* vs. *N. alba* and *N. pallida* vs. *P. cineraria* showed a 1:2 pattern (Fig. 6). This is explained by the number of genes from either *N. alba* or *P. cineraria* that are absent in our assembly (32.8 and 15.6%, respectively), and the number of *N. pallida* genes that appear duplicated in both *N. alba* and *P. cineraria* assemblies (26 and 12.4%, respectively). These patterns could be due to the fragmentation degree of both *N. alba* and *P. cineraria* assemblies (6,087 and 2,265 segments, respectively). Alternatively, these results suggest that the genome downsizing observed for *N. pallida* involves both non-coding and protein-coding regions in the genome. Although the mechanism underlying this observation could be neutral, it may have led to a specialization of *N. pallida* to its unique niche, concordant with the species being one of the two truly tropical species in the former *Prosopis* genus (Pasiecznik *et al.* 2001).

### Gene family inference and dynamics

**Fig. 7.**
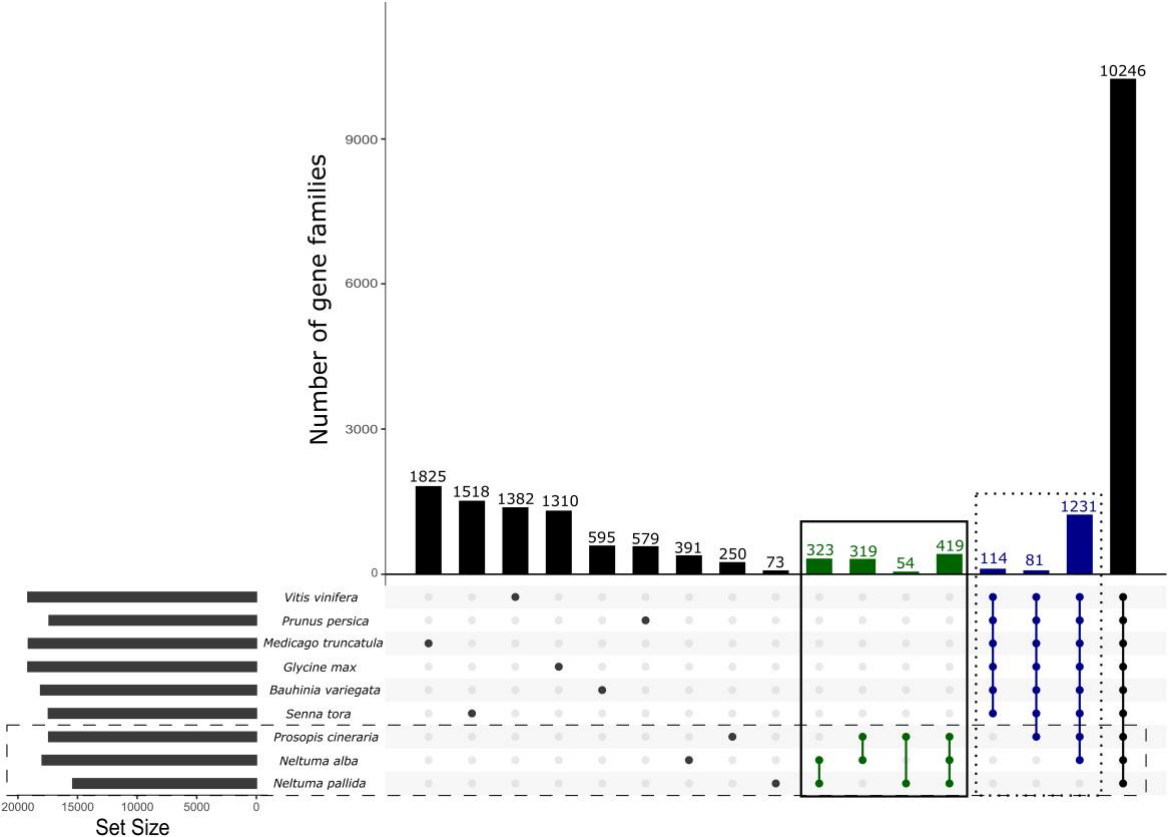
Gene families (HOGs) found by OrthoFinder specific to each species and shared among species of interest. Enclosed with a solid line are gene families exclusive to members of *Neltuma* or the former *Prosopis*. A dotted line encloses gene families present in other plant species but absent in *N. pallida*, *Neltuma*, or the former *Prosopis* genus (*Neltuma* and *P. cineraria*). The dashed line encloses the three species from the former Prosopis genus included in this study.

A total of 21,534 *N. pallida* genes were included in 14,532 gene families along other plant species. From these, 73 gene families included only *N. pallida* genes, and 875 genes remained unassigned (Fig. 7, Supplementary Table 3). A total of 1,115 gene families were present only for the *Neltuma* genus (*N. pallida* and *N. alba*) or the former *Prosopis* genus (*Neltuma* and *P. cineraria*). On the other hand, 1,231 gene families were present in all other species except *N. pallida* (Fig. 7).

**Fig. 8.**
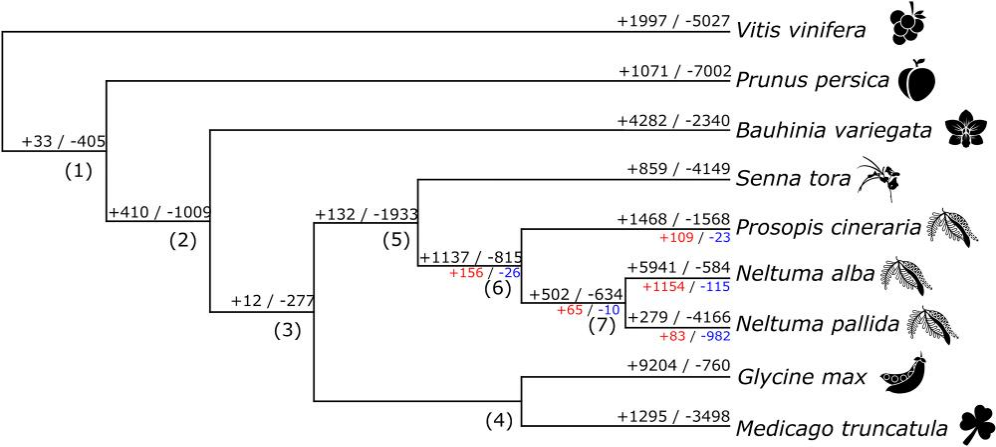
Summary of the number of expanded/contracted genes after modeling the gene family's evolution with CAFE. Each node is numbered in parenthesis for estimated gene families’ counts, changes, and probabilities in Supplementary Table 4. Significant expansions (+) and contractions (-) (branch/node specific *P*-value < 0.05) are indicated for species and nodes under the former *Prosopis* genus (*P. cineraria*, *N. alba*, and *N. pallida*).

Many *N. pallida* gene families have contracted compared with other plant species. The overall gene birth–death rate (λ) was estimated as 0.0052, while the rate of expansion for *N. pallida* was −0.28. A total of 1,065 gene families involving *N. pallida* deviated from the gene birth–death model (*P* < 0.05), 982 contracting and 83 expanding (Fig. 8 and Table 4, Supplementary Table 4). These significant contractions ranged from 1 to 22 fewer copies, while the expansions ranged from up to 13 additional copies. For expanded gene families, the number of paralogs in the *N. pallida* genome ranged from 2 to 14 copies (Supplementary Table 5). Furthermore, 47 of the expanding gene families were significant only for *N. pallida* and not for any other species included in this study (Supplementary Table 6). The complete list and annotation of genes from significantly evolving gene families is provided in Supplementary Table 7.

**Table 4.** Summary of gene family dynamics and estimated gene and family counts for the species used in the analysis.

Species	Expansions*	No change	Contractions*	Average expansion rate (*λ*)	Gene families (*n*)	Genes (*n*)
*B. variegata*	4,282 (130)	11,137	2,340 (56)	0.18	15,490	27,652
*G. max*	9,204 (420)	7,795	760 (60)	0.70	15,343	37,624
*M. truncatula*	1,295 (219)	12,966	3,498 (89)	−0.032	14,855	24,568
*N. pallida*	279 (83)	13,314	4,166 (982)	−0.28	13,126	17,961
*N. alba*	5,941 (1154)	11,234	584 (115)	0.45	14,779	30,863
*P. cineraria*	1,468 (109)	14,723	1,568 (23)	0.018	14,715	23,278
*P. persica*	1,071 (51)	9,686	7,002 (18)	−0.30	15,783	19,926
*S. tora*	859 (79)	12,751	4,149 (199)	−0.16	13,925	19,564
*V. vinifera*	1,997 (65)	10,735	5,027 (7)	−0.056	17,759	24,697

^*^Significant at branch-specific *P-value* = 0.05 in parenthesis.

The rate of expansion found for *N. pallida* is opposite to the values found both for *N. alba* (*λ* = 0.45) and for *P. cineraria* (*λ* = 0.018). Similar results were found for *N. alba*, although with fewer contracting gene families (Kong *et al.* 2023). On the other hand, a smaller positive and a large negative expansion rate was reported for *N. alba* and *P. cineraria*, respectively, elsewhere (Sudalaimuthuasari *et al.* 2022). Apart from the use of a broader species dataset in the latter study, the strikingly different results in our analysis could be due to the inclusion of *N. pallida* genome, which despite being closely related has a smaller gene content.

### Gene ontology enrichment analysis

**Fig. 9.**
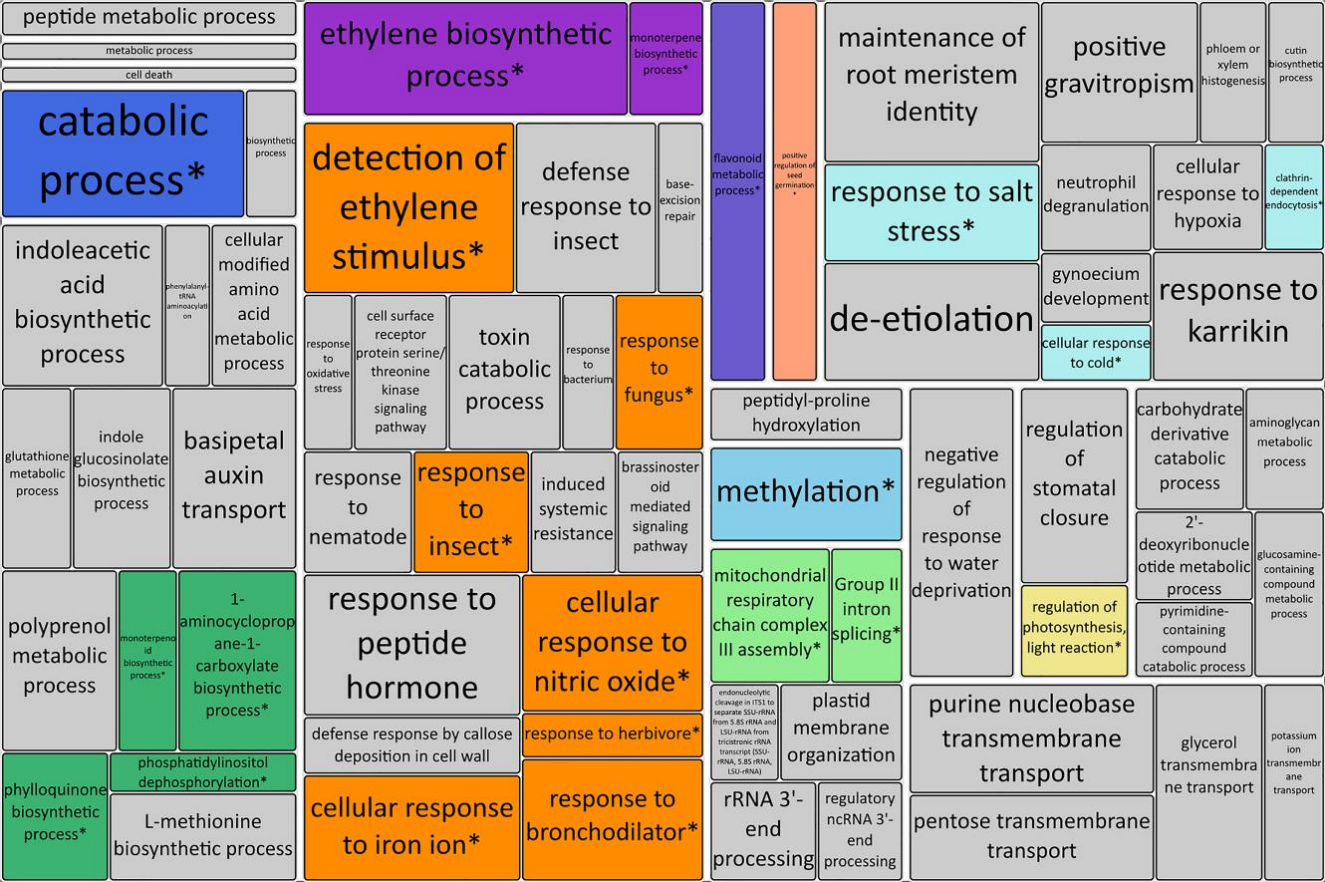
Biological process gene ontology terms significantly enriched in genes from expanding gene families involving *N. pallida*. Asterisks (*) and a non-gray colored box indicate BP GO terms also enriched in genes from gene families expanding exclusively for *N. pallida*.

In the context of a relatively small genome and a large number of contracting gene families, we explored whether the few expanding (or persisting) gene families were potentially related to the unique adaptations of *N. pallida*. A total of 155 gene ontology (GO) terms were significantly enriched (*P* < 0.01) in the expanding gene families involving *N. pallida*, including 94 biological processes (BP), 52 molecular functions (MF) (52), and 9 cellular components (Fig. 9 and Table 5, Supplementary Table 8). Furthermore, 74 GO terms were found enriched in the gene families significantly expanding only for the *N. pallida* branch (Supplementary Table 9).

**Table 5.** List of top five enriched GO terms for each category in significantly expanded gene families involving N. pallida.

Type	GO term	Description	*P* value
Biological process	GO:0009693*^a^*	ethylene biosynthetic process	9.4E-13
	GO:0009727*^a^*	detection of ethylene stimulus	5.3E-12
	GO:0010078	maintenance of root meristem identity	1.3E-11
	GO:0009056*^a^*	catabolic process	7.5E-11
	GO:0080148	negative regulation of response to water deprivation	1.1E-10
MF	GO:0022821	solute:potassium antiporter activity	3.9E-14
	GO:0008061	chitin binding	5.4E-14
	GO:0030761*^a^*	8-hydroxyquercitin 8-O-methyltransferase activity	1.1E-12
	GO:0050362	L-tryptophan:2-oxoglutarate aminotransferase activity	2.1E-12
	GO:0009815*^a^*	1-aminocyclopropane-1-carboxylate oxidase activity	2.1E-12
Cellular component	GO:0009705	plant-type vacuole membrane	1.4E-6
	GO:0009543*^a^*	chloroplast thylakoid lumen	1.5E-6
	GO:0016020	membrane	1.1E-5
	GO:0005618*^a^*	cell wall	0.00047
	GO:0005773	vacuole	0.00126

^*a*^GO terms also significantly enriched in gene families expanding only for the *N. pallida* branch.

Some of these unusually expanded gene families may explain the high capability of *N. pallida* to withstand the harsh conditions imposed by the tropical dry forest climate. *N. pallida* alternates between periods of survival during the dry season, and rapid growth when water becomes seasonally available or due to El Niño events (Salazar *et al.* 2021). Expanded gene families enriched involved in catabolic processes and regulation of photosynthesis and proteolysis, as well as responses to biotic (bacteria, fungi, insects, herbivores) and abiotic (nutrients, salt, water, cold stress) stimuli, and regulating molecules are likely related to physiological adaptations that maintain cellular homeostasis, efficient resource uptake and use and adapted immune response. On the other hand, expanded gene families related to the positive regulation of seed germination, molecule transport, and the biosynthetic pathways of various metabolites (ethylene, phylloquinone, monoterpenes, flavonoids, sesquiterpenoid) could have an important role during wet periods when growth is prioritized.

Notably, our results show an expansion of gene families involved in the ethylene biosynthesis pathway, 1-aminocyclopropane-1-carboxylate synthase (N0.HOG0021956 and N0.HOG0001203) and 1-aminocyclopropane-1-carboxylate oxidase (N0.HOG0000877 and N0.HOG0000203; Supplementary Table 7). Ethylene is a vital phytohormone known to regulate numerous processes in growth, development, and stress response in a concentration- and timing-dependent, as well as species-specific manner (Hao *et al.* 2017; Iqbal *et al.* 2017). The extreme variability in water availability to which *N. pallida* is exposed due to the pronounced bimodal seasonality of the dry forests and sporadic El Niño events may have promoted an ethylene-mediated mechanism of adaptation via the retention and expansion of genes encoding key enzymes. Other relevant expanded gene families are involved in the phenylpropanoid pathway, encoding proteins such as Cinnamoyl-CoA dehydrogenases (CAD, N0.HOG0000295), flavin adenine dinucleotide-linked oxidoreductases (N0.HOG0000034), and proteins similar to peroxidases (N0.HOG0004155 and N0.HOG0002759), which are involved in lignin and flavonoid metabolic pathways. Lastly, expansions of proteins similar to PHYLLO (N0.HOG0008712) and organic cation transporters (OCT, N0.HOG0003030) might further explain *N. pallida* remarkable adaptation to tropical dry forests.

### Conclusions

Here, we present the first chromosome-scale reference genome for the *Neltuma* genus and former *Prosopis* group (403.7 Mb, 14 pseudochromosomes and 63 scaffolds) with 51.15% of its genome being repetitive. The *N. pallida* genome assembly is of high quality as indicated by the BUSCO completeness percentage (99.2%) and N50 (26.2 Mb). Gene annotation relied on multiple sources of information with a transcriptome BUSCO assessment of completeness of 89.1%. Our results indicate that the remarkable adaptation of *N. pallida* to the conditions of the tropical dry forest climate of the desertic Peruvian coast is associated with a genome downsizing while retaining or expanding genes associated with the regulation of growth, development, and stress tolerance. However, further studies would be needed to determine if these gene expansions are linked with increased gene expression or protein/metabolite content in relevant developmental stages or under environmental stress. While there is still a large knowledge gap for the genomics of the South-American algarrobos and the disintegrated *Prosopis* group, access to the *N. pallida* genome sequence will facilitate studies underlying adaptation to extreme environments, genetic biotechnological improvement of *N. pallida*, and guide conservation efforts urgently needed in *N. pallida* native areas.

## Supplementary Material

jkae283_Supplementary_Data

## Data Availability

Additional files were submitted through the GSA submission portal. Supplementary Fig. 1 GenomeScope results for *N. pallida* genomic short-read sequencing data. Supplementary Fig. 2 Smudgeplot of *N. pallida* genomic short-read sequencing data showing the most likely ploidy. Supplementary Fig. 3 K-mer based assembly coherence evaluation using genomic short-read sequencing data before (a) and after (b) haplotig removal. Supplementary Fig. 4 Hi-C interaction heatmap generated during the scaffolding process. Supplementary Fig. 5 Synteny depths in pairwise comparisons between (a) *N. pallida* and *V. vinifera*, (b) *N. pallida* and *M. truncatula*, and (c) *N. pallida* and *G. max*. Supplementary Table 1 Genome characteristics of the species included in this study. Supplementary Table 2 Protein-coding gene characteristics of the species included in this study. Supplementary Table 3 List of Orthofinder's phylogenetic hierarchical gene families (HOGs) including *N. pallida* genes. Supplementary Table 4 Expanded CAFE results for all branches and nodes, showing the number of expansions, contractions, and gene families with no change. Supplementary Table 5 Summary of gene family changes, counts, and probabilities for all species included in this study. Supplementary Table 6 Overlapping significantly expanding gene families among the species included in this study. Supplementary Table 7 Functional annotation of genes in rapidly expanding families involving *N. pallida* (*P* < 0.05). Supplementary Table 8 Gene Ontology (GO) enrichment analysis of genes in rapidly expanding families involving *N. pallida*. Supplementary Table 9 Gene Ontology (GO) enrichment analysis of genes in rapidly expanding families only in *N. pallida*. Additional sequence, alignment, annotation and Circos files can be found in Figshare DOI: https://doi.org/10.6084/m9.figshare.25517770. Raw sequencing data are available at NCBI Sequence Read Archive BioProject PRJNA1102382. Codes used for genome assembly, annotation and comparative analyses can be found in a Github repository at https://github.com/RenatoLaTorreRamirez/Algarrobo-Genome. Supplemental material available at G3 online.
