# Supplementary figures and images for: A chromosome-level genome assembly of the Peruvian Algarrobo (Neltuma pallida) provides insights on its adaptation to its unique ecological niche
Source: G3 (Bethesda). 2024 Dec 5;15(2):jkae283. doi: 10.1093/g3journal/jkae283 (PMC11797065; doi:10.1093/g3journal/jkae283)

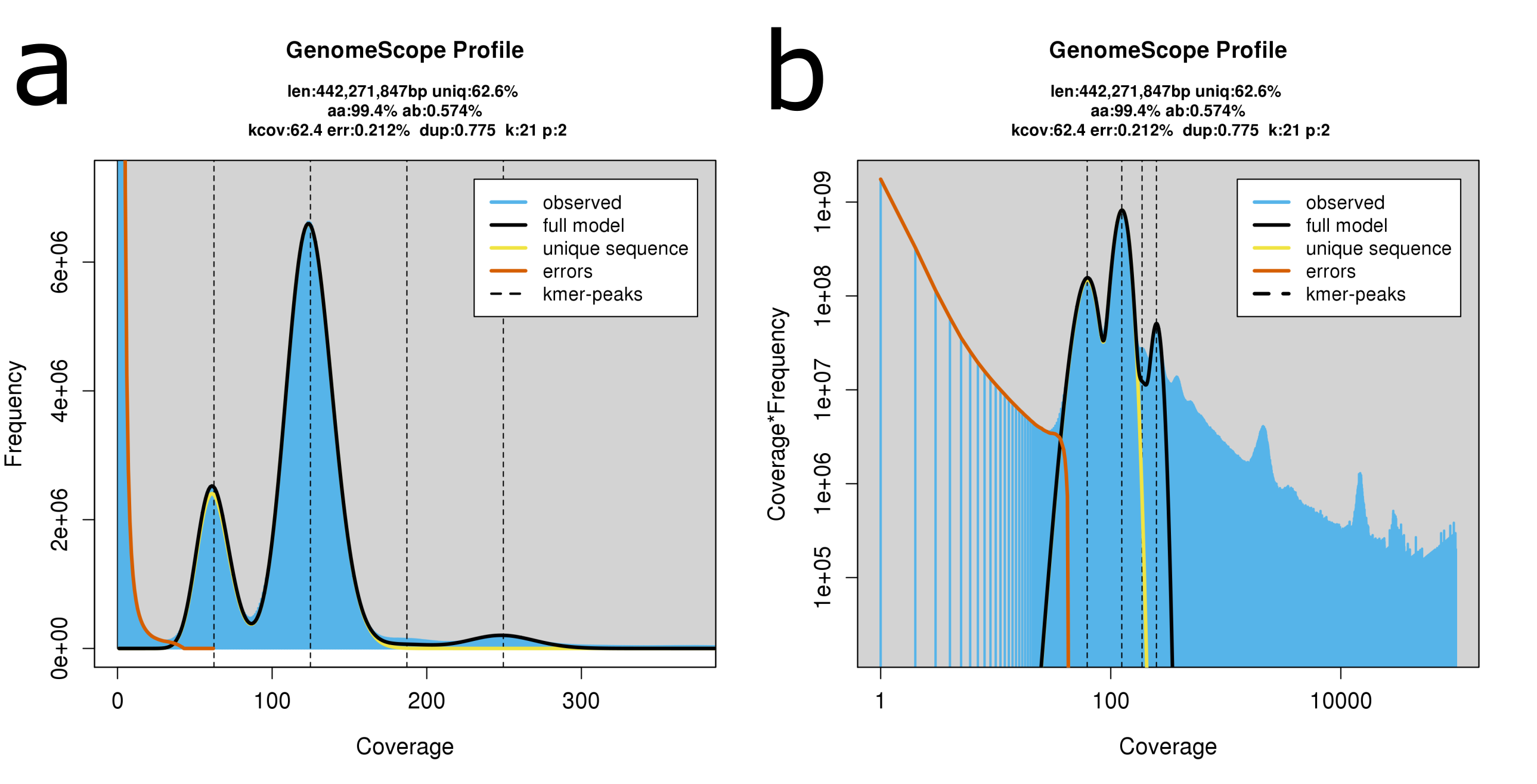

Supplement: jkae283_Supplementary_Data [file jkae283_supplementary_data.zip › Figure_S1_G3-2024-405403.tif]

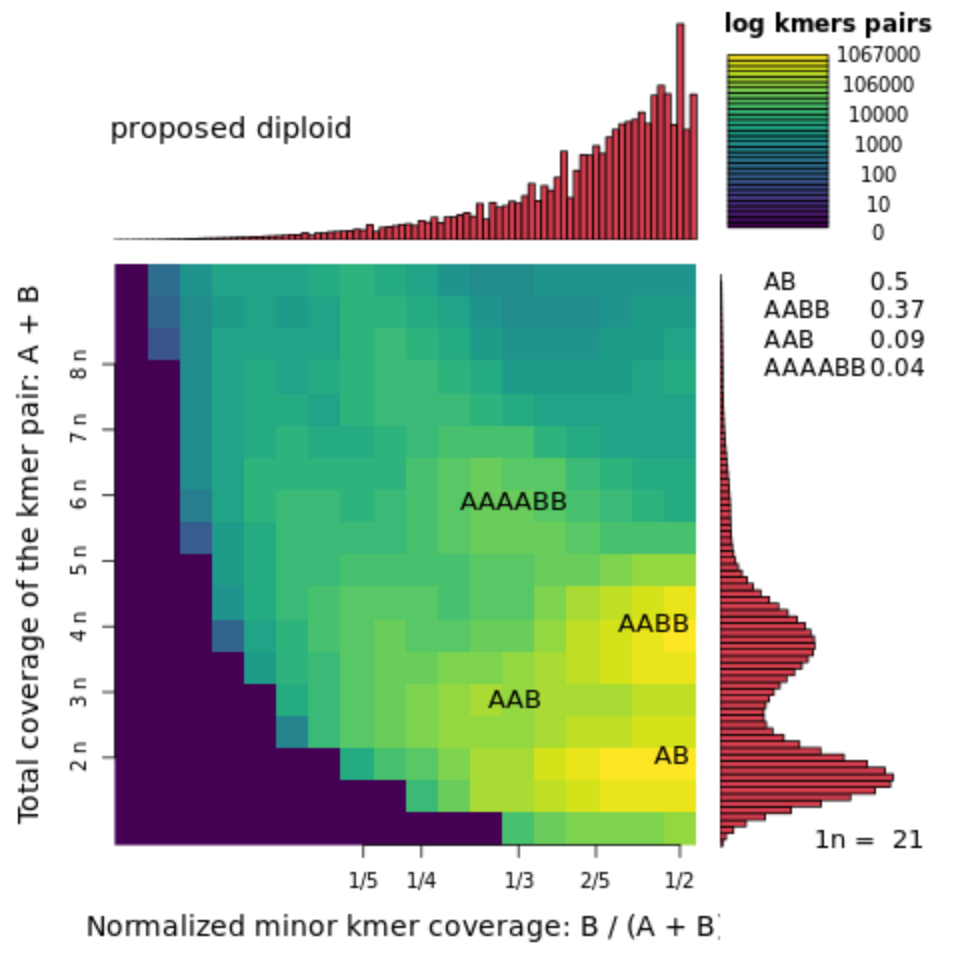

Supplement: jkae283_Supplementary_Data [file jkae283_supplementary_data.zip › Figure_S2_G3-2024-405403.tif]

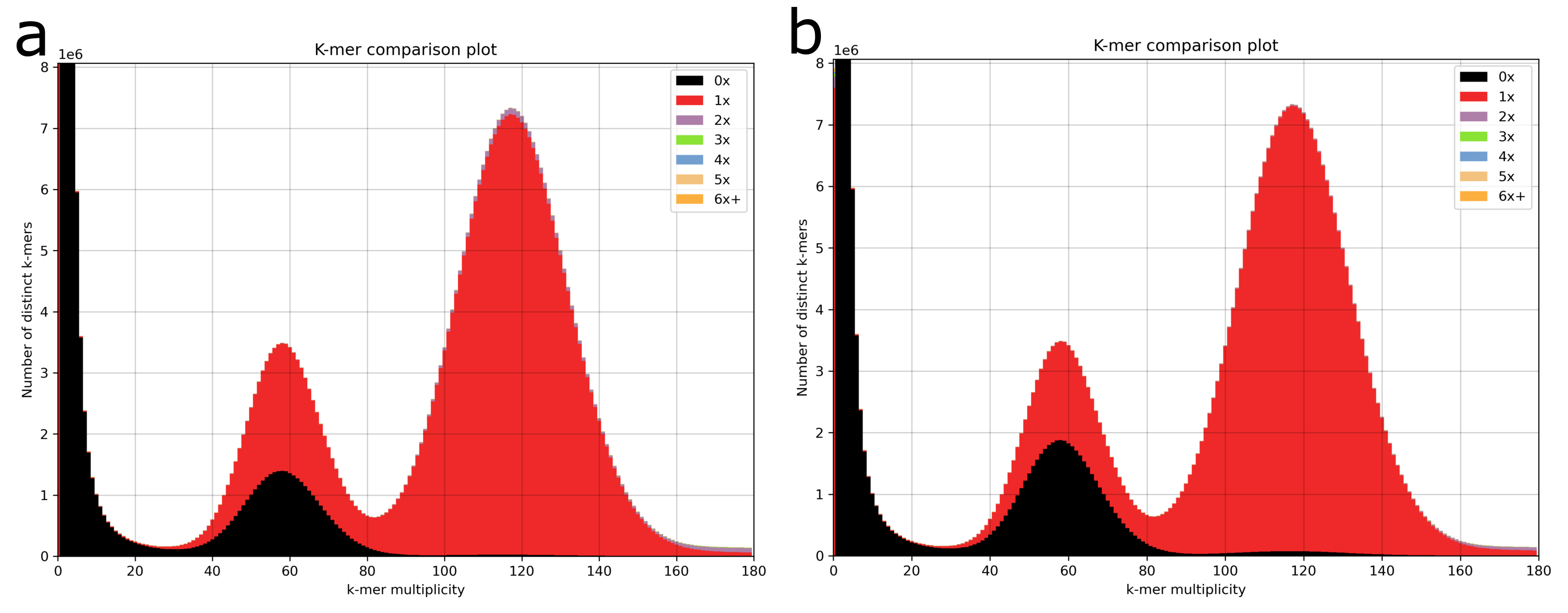

Supplement: jkae283_Supplementary_Data [file jkae283_supplementary_data.zip › Figure_S3_G3-2024-405403.tif]

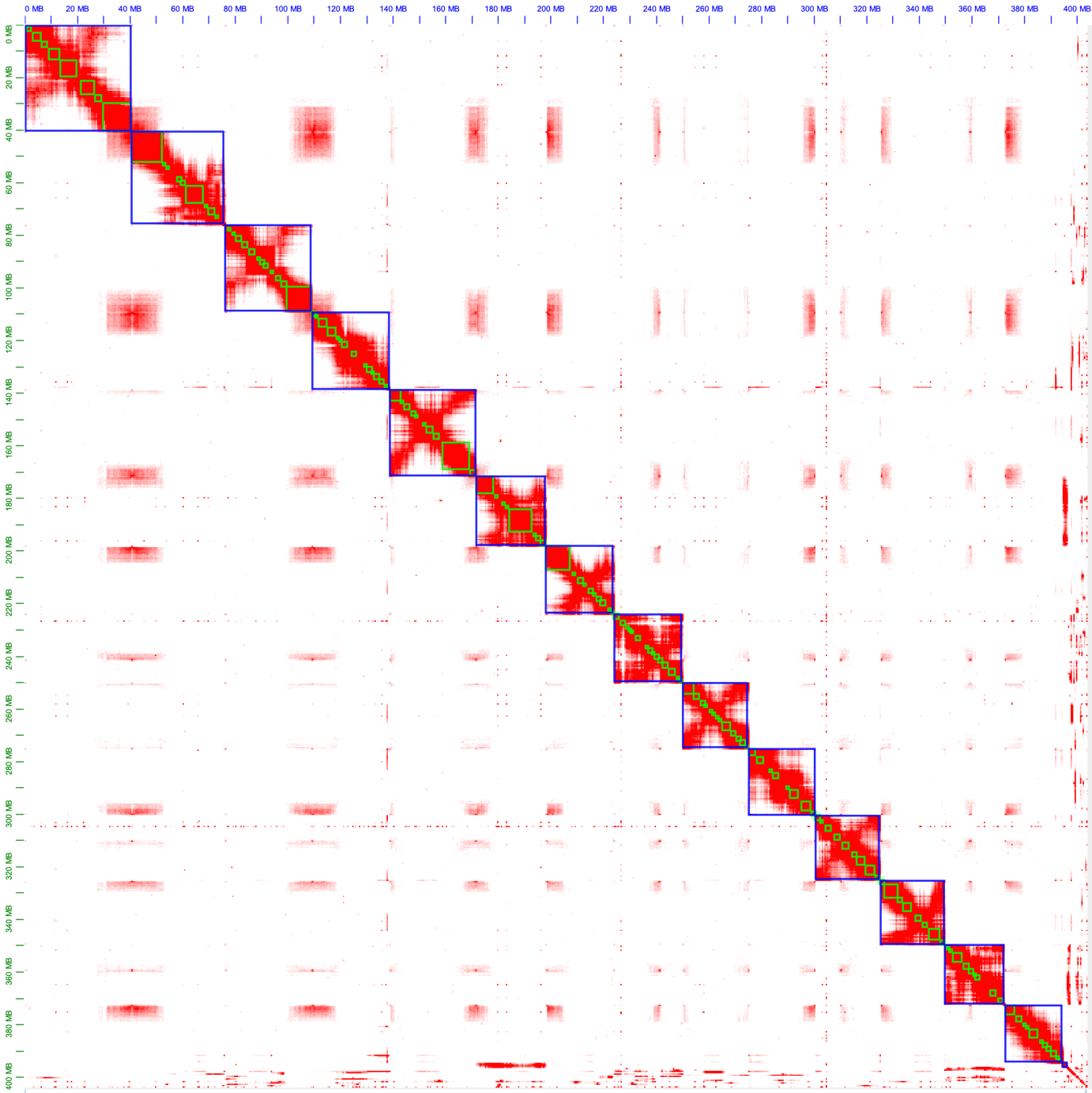

Supplement: jkae283_Supplementary_Data [file jkae283_supplementary_data.zip › Figure_S4_G3-2024-405403.tif]

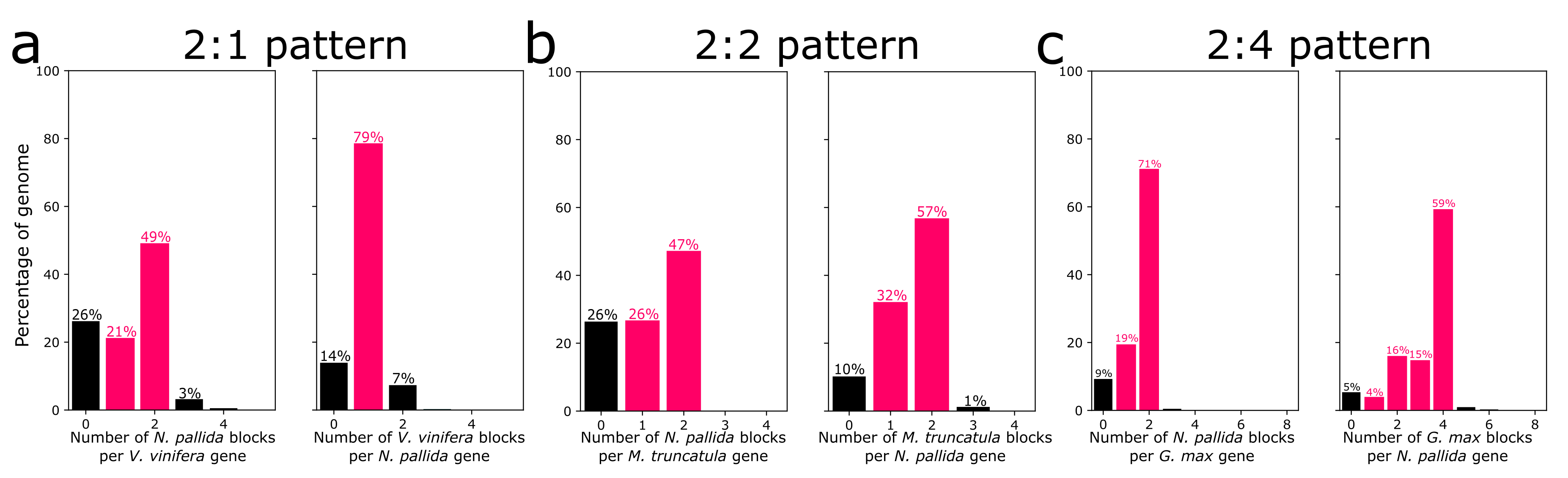

Supplement: jkae283_Supplementary_Data [file jkae283_supplementary_data.zip › Figure_S5_G3-2024-405403.tif]
